# Macrophage C3aR1 Mediates Sepsis‐Induced Myocardial Injury by Triggering Neutrophil Necroptosis

**DOI:** 10.1111/jcmm.71145

**Published:** 2026-04-19

**Authors:** Jianbo Xu, Zhilei He, Rubing Zhang, Chaonan Peng, Ziteng Cai, Xinyue Zhao, Weiqun Wang

**Affiliations:** ^1^ Key Laboratory of Microecology‐Immune Regulatory Network and Related Diseases, Basic Medical College Jiamusi University Jiamusi Heilongjiang China; ^2^ Department of Critical Care Medicine Hanchuan People's Hospital Xiaogan Hubei Province China; ^3^ Teaching and Research Section of Physiology, the Basic Medical College Jiamusi University Jiamusi Heilongjiang China; ^4^ State Key Laboratory of Neurology and Oncology Drug Development Nanjing Jiangsu China

**Keywords:** C3aR1, immune inflammation, macrophage polarisation, neutrophil necroptosis, sepsis‐induced myocardial injury

## Abstract

Sepsis‐induced myocardial injury (SIMI) is a leading cause of mortality in critically ill patients, driven by dysregulated immune‐inflammation responses. Although macrophages and neutrophils are key players in this process, the mechanisms governing their crosstalk in SIMI remain unclear. Here, we demonstrate that the complement C3a receptor 1 (C3aR1) critically mediates this interaction. Using a cecal ligation and puncture (CLP)‐induced SIMI rat model and an in vitro co‐culture system with THP‐1‐derived macrophages, HL‐60 cells and AC16 cardiomyocytes, we show that C3aR1 promotes macrophage M1 polarisation via the TLR4/NF‐κB pathway. The activated M1 macrophages subsequently trigger neutrophil necroptosis, leading to the release of chemokines and establishing a self‐amplifying inflammatory loop from M1 polarisation to neutrophil necroptosis and cardiomyocyte injury, ultimately resulting in cardiac dysfunction. Cardiac‐specific knockdown of C3aR1 in vivo attenuated myocardial damage, reduced inflammatory cell infiltration and improved cardiac function. Our findings identify C3aR1 as a key molecular hub orchestrating macrophage‐neutrophil crosstalk in SIMI and highlight its potential as a therapeutic target for mitigating sepsis‐induced cardiac complications.

## 1 Introduction

Sepsis is a systemic inflammatory response triggered by infection that can lead to septic shock and multiple organ failure [1, 2]. Statistical data indicate that there are approximately 30 million sepsis patients globally, among whom 40%–50% develop concurrent myocardial dysfunction, characterised by myocardial tissue damage and functional impairment. This represents a major factor contributing to mortality in septic patients [3, 4]. Although its pathophysiological mechanisms are complex and multifactorial, dysregulated immune‐inflammatory responses are considered central to driving myocardial injury [5, 6]. Recent deep immunophenotyping analysis has revealed significant remodelling of innate immune cell subsets (such as natural killer cells and dendritic cells) in septic patients [7]. Meanwhile, plasma proteomic studies have demonstrated that sepsis mortality‐associated proteins are enriched in immune response and complement activation pathways, highlighting the central role of innate immunity [8]. Macrophages and neutrophils, as primary effector cells of innate immunity, contribute collectively through aberrant activation, recruitment and intricate intercellular communication to form the inflammatory microenvironment underlying sepsis‐induced myocardial injury [9]. However, existing research has largely focused on the isolated functions of individual cell types and the specific molecular mechanisms by which these two cell types cooperate within myocardial lesions to amplify inflammatory signals remain to be fully elucidated.

Necroptosis is a recently identified regulated cell death pathway involved in intestinal homeostasis and inflammation. It represents a form of necrotic cell death initiated by death receptors (such as TNF‐α receptor 1) and mediated by receptor‐interacting protein kinases RIP1 and RIP3 [10]. In sepsis, necroptosis is activated and exacerbates myocardial inflammatory responses and cell death through the release of pro‐inflammatory factors [11]. Studies have found upregulated expression of key necroptosis markers in the myocardial tissue of septic patients, directly contributing to cardiomyocyte death and dysfunction [12]. Nevertheless, the precise role of necroptosis in the dysregulated immune inflammation characteristic of sepsis‐induced myocardial injury remains incompletely understood.

In the immune storm triggered by sepsis, macrophages act as the “command center” of the inflammatory response [13]. Their functional plasticity allows them to differentiate into pro‐inflammatory M1 or anti‐inflammatory M2 phenotypes in response to different microenvironmental signals. Studies indicate that in sepsis‐induced myocardial injury models, M1‐polarised macrophages are significantly increased in cardiac tissue and positively correlate with decreased cardiac function [14, 15]. M1‐polarised macrophages directly induce cardiomyocyte injury and mitochondrial dysfunction by releasing pro‐inflammatory factors (such as TNF‐α and IL‐1β) and reactive oxygen species (ROS) [16, 17].

Meanwhile, neutrophils, as the most abundant type of immune cells, constitute a crucial line of immune defence. Excessive infiltration and degranulation of neutrophils can trigger cardiomyocyte apoptosis and tissue destruction [18, 19, 20]. Furthermore, the generation of ROS is believed to be involved in the process of necroptosis, although its specific sources and mechanisms of action are not fully elucidated [21, 22]. Tissue macrophages play a significant role in neutrophil recruitment [23, 24]; however, whether macrophages recruit neutrophils and induce their necroptosis remains to be explored.

C3aR1 is a G protein‐coupled receptor expressed in various immune cells that regulates immune cell activation and function by binding to C3a [25]. Studies have shown that C3aR1 signalling participates in immune regulation through multiple mechanisms. In tumour immunity, it interacts with LFA‐1 to induce conformational changes, inhibiting activated NK cell migration into the tumour microenvironment [26]. In humoral immunity, C3a stimulates ILC2s to secrete IL‐5, supporting B1 cell activation and C3ar1^−/−^ mice exhibit a significantly reduced antibody response [27]. The role of C3aR1 is particularly prominent in tissue injury models. In renal ischemia/reperfusion injury, the C3a/C3aR axis exacerbates tissue damage by promoting neutrophil recruitment and NETs formation; knockout of C3aR or blockade of NETs improves renal function [28]. In silicosis, this axis is persistently activated in M1 macrophages and its inhibition reduces macrophage recruitment and M1 polarisation via the CCL2‐CCR2 pathway [29]. Furthermore, in rheumatoid arthritis, C3a derived from synovial fibroblasts activates macrophage C3aR1, promoting a type I interferon response that feedback‐activates fibroblasts, forming a pro‐inflammatory positive feedback loop, suggesting a potential role for C3aR1‐mediated macrophage activation in influencing neutrophil recruitment [30]. In the context of sepsis, the central role of C3aR1 is increasingly evident. Research confirms that C3aR1 expression is elevated in the plasma of sepsis patients, with single‐cell sequencing identifying its predominant enrichment in macrophages [31]. In sepsis‐induced acute lung injury, the C3a‐C3aR1 axis induces pyroptosis in pulmonary endothelial cells by activating the NLRP3/caspase‐1 pathway and inhibition of this axis significantly attenuates lung injury [32]. Given its central role in regulating immune cell function and inflammatory signalling, C3aR1 has been identified as a potential interventional target for sepsis by multiple studies [33]. However, the mechanism by which C3aR1 regulates macrophage‐neutrophil interactions in sepsis‐induced myocardial injury remains unclear.

This study aims to investigate the molecular mechanisms by which C3aR1 regulates the interaction between macrophages and neutrophils in sepsis‐induced myocardial injury. Through in vivo and in vitro experiments, we seek to systematically validate the complete pathological pathway from C3aR1 activation driving macrophage M1 polarisation to inducing neutrophil necroptosis, ultimately leading to cardiomyocyte injury. This research is expected not only to elucidate the immunopathological mechanisms of sepsis‐induced myocardial injury by establishing an organic connection between complement activation, macrophage polarisation and neutrophil death modalities, but also to provide a crucial theoretical foundation for developing immune intervention strategies targeting key molecules such as C3aR1.

## 2 Materials and Methods

### 2.1 Materials

#### 2.1.1 Experimental Animals

Male Sprague Dawley (SD) rats (weighing 220–250 g, aged 4–6 weeks) of specific pathogen‐free (SPF) grade were purchased from Zhiyuan Biotechnology Co. Ltd. (Guangdong, China). SD rats were selected due to their well‐characterised immune responses to septic challenge and their extensive use in cardiovascular research, providing reliable reproducibility for modelling sepsis‐induced myocardial injury. All rats were housed in an SPF‐grade animal room maintained at a temperature of (22°C ± 2°C) and relative humidity of (50% ± 5%), under a 12‐h light/dark cycle. The animals had free access to standard laboratory chow and sterile drinking water. After 1 week of acclimatisation, the experiments were initiated.

#### 2.1.2 Cells

The human monocytic leukaemia cell line (THP‐1), human promyelocytic leukaemia cell line (HL‐60) and human cardiomyocyte cell line (AC16) were all purchased from the Chinese Academy of Sciences Shanghai Cell Bank (Shanghai, China). THP‐1 cells are widely used as a human macrophage model due to their ability to differentiate into macrophage‐like cells, closely mimicking primary human macrophage functions, including polarisation responses. HL‐60 cells can be differentiated into neutrophil‐like cells, serving as a well‐established in vitro model for studying human neutrophil biology and cell death pathways. AC16 cells are a human cardiomyocyte cell line that retains essential cardiac‐specific characteristics, including the expression of cardiac markers and responsiveness to stress stimuli, making them a relevant model for investigating human myocardial injury mechanisms. This combination of human‐derived cell lines enables translational relevance by allowing mechanistic studies in human immune and cardiac cells under controlled conditions.

#### 2.1.3 Major Equipment

Real‐time quantitative PCR system (Bio‐Rad CFX96, USA), flow cytometer (BD FACSCanto II, USA), transmission electron microscope (TEM; JEOL JEM‐1400, Japan), small animal cardiac ultrasound imaging system (VisualSonics Vevo 2100, USA), western blot (WB) electrophoresis and transfer systems (Bio‐Rad Mini‐PROTEAN Tetra, USA), fluorescence microscope (Olympus IX73, Japan), microplate reader (Thermo Scientific Multiskan FC, USA), CO_2_ incubator (Thermo Scientific 3111, USA), high‐speed centrifuge (Eppendorf 5810R, Germany) and tissue embedding machine/microtome (Leica RM2235, Germany).

### 2.2 Methods

#### 2.2.1 Construction and Grouping of Rat Sepsis‐Induced Myocardial Injury Model

The cecal ligation and puncture (CLP) method was used to establish a sepsis‐induced myocardial injury model in SD rats. After being anaesthetised by intraperitoneal injection of 1% sodium pentobarbital solution (40 mg/kg), the rats were placed in a supine position and securely fixed. A midline abdominal incision (approximately 2 cm in length) was made to expose the cecum. The distal one‐third of the cecum was ligated with 4–0 silk suture and perforated once using a 22‐gauge needle. A small amount of faecal content was gently extruded to ensure the patency of the perforation. The cecum was then returned to the abdominal cavity and the abdominal wall was sutured layer by layer. In the sham group (sham‐operated group), the abdominal cavity was opened and the cecum was exposed but neither ligated nor punctured before being returned to the abdomen.

A total of 60 SD rats were randomly assigned to 4 groups (*n* = 5 per group, with triplicate validation experiments; total sample size was adjusted based on statistical power calculation): Sham group: Sham operation + normal saline injection. Model group: CLP model + normal saline injection. si‐C3aR1 group: CLP model + localised myocardial injection (Cardiac in situ injection of lentiviruses was referenced to previous methods [34, 35]. Using a Hamilton syringe, 3–5 injection sites were selected in areas of thicker myocardium, with 10–15 μL viral solution injected per site; administered 1 week prior to surgery). si‐NC group: CLP model + localised myocardial injection. All procedures were approved by the Institutional Animal Care and Use Committee.

#### 2.2.2 Transcriptomic Sequencing and Analysis Methods

##### 2.2.2.1 Transcriptome Analysis

Total RNA was extracted from myocardial tissues of the sham‐operated and model groups using TRIzol reagent. RNA integrity and concentration were assessed using the Agilent Bioanalyzer 2100 system, with RNA Integrity Number (RIN > 7.0) required for library construction. mRNA was isolated from total RNA using oligo(dT)‐conjugated magnetic beads and fragmented into approximately 200–300 bp fragments. The fragmented mRNA was reverse‐transcribed into cDNA using SuperScript II reverse transcriptase. Following double‐stranded cDNA synthesis, end repair, A‐tailing and adapter ligation were performed according to the Illumina standard protocol. PCR amplification was performed to enrich the cDNA libraries. The library quality was validated using the Agilent 2100 Bioanalyzer and sequencing was performed on the Illumina Novaseq 6000 platform.

For bioinformatics analysis, raw sequencing data were first processed using Cutadapt (v1.9) to remove adapter sequences, poly‐N tails and low‐quality bases (Q‐score < 20). Quality control of clean reads was assessed using FastQC (v0.11.7) to verify base quality distribution, GC content and sequence duplication levels. Clean reads were aligned to the *Rattus norvegicus* reference genome (Ensembl release 98) using HISAT2 (v2.1.0) with default parameters. Gene expression levels were quantified using StringTie (v1.3.3b) and expression abundance was normalised to fragments per kilobase of transcript per million mapped reads FPKM. Only genes with FPKM > 1 in at least one group were retained for downstream analysis.

##### 2.2.2.2 Immune Cell Infiltration Analysis

To evaluate the immune cell composition in myocardial tissue, the CIBERSORT algorithm was employed. Based on gene expression profile data, the relative proportions of 22 immune cell subtypes were inferred through deconvolution analysis using a linear support vector regression model. The analysis was performed with 100 permutations for statistical significance and samples with CIBERSORT output *p* < 0.05 were considered reliable for downstream interpretation. Additionally, Spearman correlation analysis was systematically applied to assess relationships between different immune cell types, with correlation coefficients and *p*‐values calculated using the cor.test function in R.

##### 2.2.2.3 Differential Expression Analysis

Differentially expressed analysis between sham‐operated and model groups was performed using the ‘limma’ package (v3.56.2) in R (v4.1.0). Raw counts were normalised using the voom transformation, which estimates the mean–variance relationship and generates precision weights for linear modelling. Genes with |log_2_FC| > 1 and Benjamini‐Hochberg adjusted *p*‐value < 0.05 were defined as significantly differentially expressed genes (DEGs). The ‘complexheatmap’ package was used to generate heatmaps visualising expression patterns of DEGs and their hierarchical clustering relationships among samples.

##### 2.2.2.4 KEGG and GO Enrichment Analysis

To further elucidate the potential biological functions and related signalling pathways of the DEGs, Gene Ontology (GO) and Kyoto Encyclopaedia of Genes and Genomes (KEGG) pathway were performed using the clusterProfiler R package (v4.8.1). GO analysis covered three categories: Biological Process (BP), Cellular Component (CC) and Molecular Function (MF). KEGG enrichment identified metabolic and signal transduction pathways associated with the DEGs. Enrichment significance was determined using a hypergeometric test, with *p*‐values adjusted by the Benjamini–Hochberg method to control the False Discovery Rate (FDR). Enriched terms with adjusted *p* < 0.05 and FDR < 0.05 were considered statistically significant. Results were visualised using bubble charts and bar plots generated with the ggplot2 package in R.

#### 2.2.3 RT‐qPCR Analysis

Fresh myocardial tissue (approximately 50 mg) or harvested cells were homogenised in TRIzol (TaKaRa, Japan) reagent and total RNA was extracted according to the manufacturer's instructions. RNA purity (A260/A280 ratio of 1.8–2.0) and concentration were determined using a Nanodrop 2000 spectrophotometer. Subsequently, 1 μg of total RNA was reverse‐transcribed into cDNA using a commercial reverse transcription kit. RT‐qPCR was performed using the synthesised cDNA as the template. The reaction mixture (20 μL) contained: 10 μL of SYBR Premix Ex Taq II (TaKaRa, Japan), 0.8 μL each of forward and reverse primers (10 μmol/L), 2 μL of cDNA and 6.4 μL of ddH_2_O. The amplification protocol consisted of: initial denaturation at 95°C for 30 s; 40 cycles of denaturation at 95°C for 5 s and annealing/extension at 60°C for 30 s; followed by melt curve analysis (95°C for 15 s, 60°C for 1 min and 95°C for 15 s). GAPDH was used as the internal reference gene and the relative expression level of C3aR1 mRNA was calculated using the 2^−ΔΔCt^ method. All primers were designed and synthesised by Sangon Biotech (Shanghai, China) and their sequences are listed in Table 1.

**TABLE 1:** Primer sequences.

Genes	Upper primers (5′‐3′)	Downstream primers (5′‐3′)
C3AR1	CAGGACAAAGCCCCCTCAAA	AGGAGATGGCAAGATCCTCAG
CD206	GGAACTGACCCAGTGGTTGT	AGGCGTCCATTCGTCTTCTT
iNOS	ACATCAGGTCGGCCATCACT	CCAGTAGCTGCCACACCATC
Arg1	CTCCAAGCCAAAGTCCTTAGAG	AGGAGCTGTCATTAGGGACATC
TGF‐β	GCAACAACGCCATCTATGAG	GTTGTACAAAGCGAGCACCG
CD86	CACACTGGATAACGGCTACAC	CAGGTAAACGCATCCCAAAG
TNF‐α	AGCCCCCAGTCTGTATCCTT	CTCCCTTTGCAGAACTCAGG
GAPDH	GGAGCGAGATCCCTCCAAAAT	GGCTGTTGTCATACTTCTCATGG

#### 2.2.4 Rats Echocardiography

At 24 h post‐surgery, rats were anaesthetised (1% sodium pentobarbital solution, 40 mg/kg) and cardiac function was evaluated using a small animal ultrasound system (30 MHz). Left ventricular long‐axis and short‐axis views were obtained to measure left ventricular ejection fraction (LVEF%) and left ventricular fractional shortening (LVFS%). Measurements were repeated three times for each rat and the average values were calculated.

#### 2.2.5 Histopathological Examination of Myocardial Tissue (HE Staining)

Following echocardiography, rats were euthanised and left ventricular myocardial tissues (approximately 5 mm × 5 mm × 2 mm) were collected. The tissues were fixed in 4% paraformaldehyde for 24 h, then dehydrated through a graded ethanol series, cleared in xylene and embedded in paraffin. Sections were cut at a thickness of 4 μm. After deparaffinisation and rehydration, the sections were stained with haematoxylin for 5 min, differentiated in hydrochloric acid‐alcohol for 30 s and counterstained with eosin for 2 min. Subsequently, the sections were dehydrated, cleared and mounted with neutral balsam. Pathological changes in the myocardial tissues were observed under a light microscope.

#### 2.2.6 Flow Cytometry Detection

Cells were collected from rats in each group and digested with Collagenase IV to prepare single‐cell suspensions. The suspensions were stained with fluorescently labelled antibodies against CD86 (Abcam, Cat# ab64693) and CD206 (Abcam, Cat# ab25377). All samples were incubated at room temperature in the dark, followed by PBS washing. The M1/M2 macrophage ratio was then analysed using a flow cytometer.

#### 2.2.7 ELISA for Serum and Cellular Cytokine/Chemokine Levels

Blood samples or cell culture supernatant were collected from the abdominal aorta of rats and centrifuged at 3000 r/min for 15 min at 4°C. The resulting serum supernatant was collected and stored at −80°C for subsequent analysis. Following the manufacturer's instructions of respective ELISA kits (MLBio, Shanghai), the concentrations of troponin I (cTn‐I), brain natriuretic peptide (BNP), as well as cytokines and chemokines including IL‐6, IL‐10, CCL2, CCL3, CXCL1 and CXCL2 in myocardial tissue homogenates were measured. The absorbance (OD value) at 450 nm wavelength was determined using a microplate reader and the concentrations of target molecules in the samples were calculated based on the standard curves.

#### 2.2.8 Immunofluorescence Staining

Tissue sections were deparaffinised and rehydrated, followed by antigen retrieval using citrate buffer for 15 min. For cell samples, fixation and permeabilisation were performed. Endogenous peroxidase activity was blocked with 3% H_2_O_2_ for 10 min, followed by three washes with PBS. After blocking with 5% BSA for 30 min, the samples were incubated with primary antibodies at 4°C overnight. Following three PBS washes, the samples were incubated with corresponding fluorescent secondary antibodies at room temperature for 1 h in the dark. Nuclei were counterstained with DAPI for 5 min. After mounting, the samples were observed under a fluorescence microscope. Quantitative analysis of the target protein‐to‐nuclear fluorescence intensity ratio was performed using ImageJ software.

#### 2.2.9 Western Blot Analysis

Primary antibodies (all from Proteintech, Wuhan, China): IκBα (10268‐1‐AP), p‐IκBα (Ser32/36, 82349‐1‐RR), NF‐κB p65 (10745‐1‐AP), p‐NF‐κB p65 (Ser536, 80379‐2‐PBS), MLKL (19535‐1‐AP), p‐MLKL (Ser358, 82090‐2‐RR), TLR4 (19811‐1‐AP), RIPK1 (29932‐1‐AP), p‐RIPK1 (Ser166, 28252‐1‐AP), RIPK3 (29080‐1‐AP), GAPDH (60004‐1‐Ig). For C3AR1 (Cat. AF3165, R&D Systems, USA) and p‐RIPK3 (Ser227, Cat. 93654S, CST, USA). Myocardial tissues (50 mg) or harvested cells from each group were homogenised and lysed using RIPA (Beyotime Biotechnology, Shanghai) lysis buffer containing protease inhibitors on ice. The lysates were centrifuged and the supernatants were collected. Protein concentrations were determined using the BCA (Beyotime Biotechnology, Shanghai) assay, followed by denaturation. The proteins were separated by SDS‐PAGE using 10%–12% separating gels and subsequently transferred onto PVDF membranes. After blocking with 5% skim milk for 2 h, the membranes were incubated with primary antibodies at 4°C overnight. Following three washes with TBST, the membranes were incubated with HRP‐conjugated secondary antibodies at room temperature for 1 h. Protein bands were visualised using ECL detection and the grayscale ratio of target proteins to GAPDH was quantified using ImageJ software.

#### 2.2.10 Cell Culture and Grouping

Human monocytic leukaemia cells (THP‐1) were first differentiated into macrophages using 100 ng/mL phorbol 12‐myristate 13‐acetate for 48 h. After differentiation, the THP‐1‐derived macrophages were divided into 5 groups (*n* = 5 per group) with the following treatments and all groups were cultured for 24 h: Control (CK) group: No transfection or stimulation; cells were cultured in complete RPMI‐1640 medium (supplemented with 10% foetal bovine serum (FBS) and 1% penicillin–streptomycin solution) only. Model group: No transfection; cells were treated with 100 ng/mL LPS (MCE) and 20 ng/mL IFN‐γ to induce M1 macrophage polarisation. si‐C3aR1 group: Cells were transfected with small interfering RNA targeting C3aR1 using Lipofectamine 3000 according to the manufacturer's protocol. At 24 h post‐transfection, cells were treated with 100 ng/mL LPS and 20 ng/mL IFN‐γ. si‐NC group: Cells were transfected with negative control siRNA using the same method as the si‐C3aR1 group. At 24 h post‐transfection, cells were treated with 100 ng/mL LPS and 20 ng/mL IFN‐γ. si‐C3aR1 + NF‐κB‐act group (NF‐κB activator 1, MCE): Cells were first transfected with si‐C3aR1 (same as the si‐C3aR1 group). At 24 h post‐transfection, cells were pre‐treated with 0.90 μmol/L NF‐κB activator 1 for 1 h, followed by co‐treatment with 100 ng/mL LPS and 20 ng/mL IFN‐γ.

Human promyelocytic leukaemia cells (HL‐60) were divided into 4 groups, which corresponded to the si‐NC, Model, si‐C3aR1 and si‐C3aR1 + NF‐κB‐act groups of THP‐1‐derived macrophages (*n* = 5 per group). For each group, HL‐60 cells were cultured in a mixture of conditioned medium (collected from the corresponding THP‐1‐derived macrophage group at 24 h post‐treatment) and fresh RPMI‐1640 complete medium (1:1 volume ratio). All HL‐60 cell groups were incubated for 24 h under standard culture conditions (37°C, 5% CO_2_).

Human cardiomyocyte cells (AC16) were co‐cultured with HL‐60 cells using a Transwell insert system to avoid direct cell contact. AC16 cells were seeded in the lower chamber of 6‐well plates at a density of 5 × 10^4^ cells/well, while HL‐60 cells (from the 4 aforementioned groups) were seeded in the upper Transwell insert at a density of 1 × 10^5^ cells/well. The co‐culture system used DMEM high‐glucose complete medium (supplemented with 10% FBS and 1% penicillin–streptomycin solution) and all groups were cultured for 24 h (37°C, 5% CO_2_) before subsequent experiments. The siRNA targeting rat C3aR1 (si‐C3aR1) and a non‐targeting negative control siRNA (si‐NC) were designed and synthesised by Sangon Biotech (Shanghai, China). The siRNA sequences are as follows in Table 2.

**TABLE 2:** siRNA sequence.

Genes	Sense strand (5′‐3′)	Antisense strand (5′‐3′)
si‐C3aR1	GCAAGCUGACCUGAGUAU	AUACUCAUGGUCAGCUUGC
Si‐NC	UUCUCCGAACGUGUCACU	ACGUGACACGUUCGGAGAA

#### 2.2.11 TEM Observation of Necroptotic Morphology in HL‐60 Cells

HL‐60 cells from the four experimental groups were fixed with 2.5% glutaraldehyde followed by 1% osmium tetroxide. After graded dehydration and epoxy resin embedding, ultrathin sections were prepared and stained. The characteristic ultrastructural morphology of necroptosis, including plasma membrane disruption and organelle swelling, was examined using transmission electron microscopy.

#### 2.2.12 Detection of ROS Levels Using Fluorescent Probe

AC16 cells co‐cultured with HL‐60 cells in a Transwell system were incubated with the DCFH‐DA fluorescent probe at 37°C for 30 min. After washing off unbound probe with PBS, fluorescence intensity was measured using either flow cytometry or fluorescence microscopy to reflect intracellular ROS levels.

#### 2.2.13 Immunofluorescence Staining for MYO and Troponin

AC16 cells from the respective groups were fixed and blocked. Subsequently, the cells were incubated overnight at 4°C with primary antibodies against myoglobin (MYO) and troponin, followed by incubation with fluorescent secondary antibodies at room temperature for 1 h in the dark. Nuclei were stained with DAPI and the staining patterns were visualised under a fluorescence microscope.

#### 2.2.14 Statistical Analysis

Data analysis was performed using SPSS 26.0 software. One‐way ANOVA was used for group comparisons (after confirming normality and homogeneity of variance), followed by Tukey's post hoc test. Results are presented as mean ± standard deviation. GraphPad Prism 9.0 was used for data visualisation. A *p*‐value < 0.05 was considered statistically significant.

## 3 Results

### 3.1 Analysis of Myocardial Immune Microenvironment Remodelling and C3aR1 Expression Characteristics in Septic Rats

**FIGURE 1:**
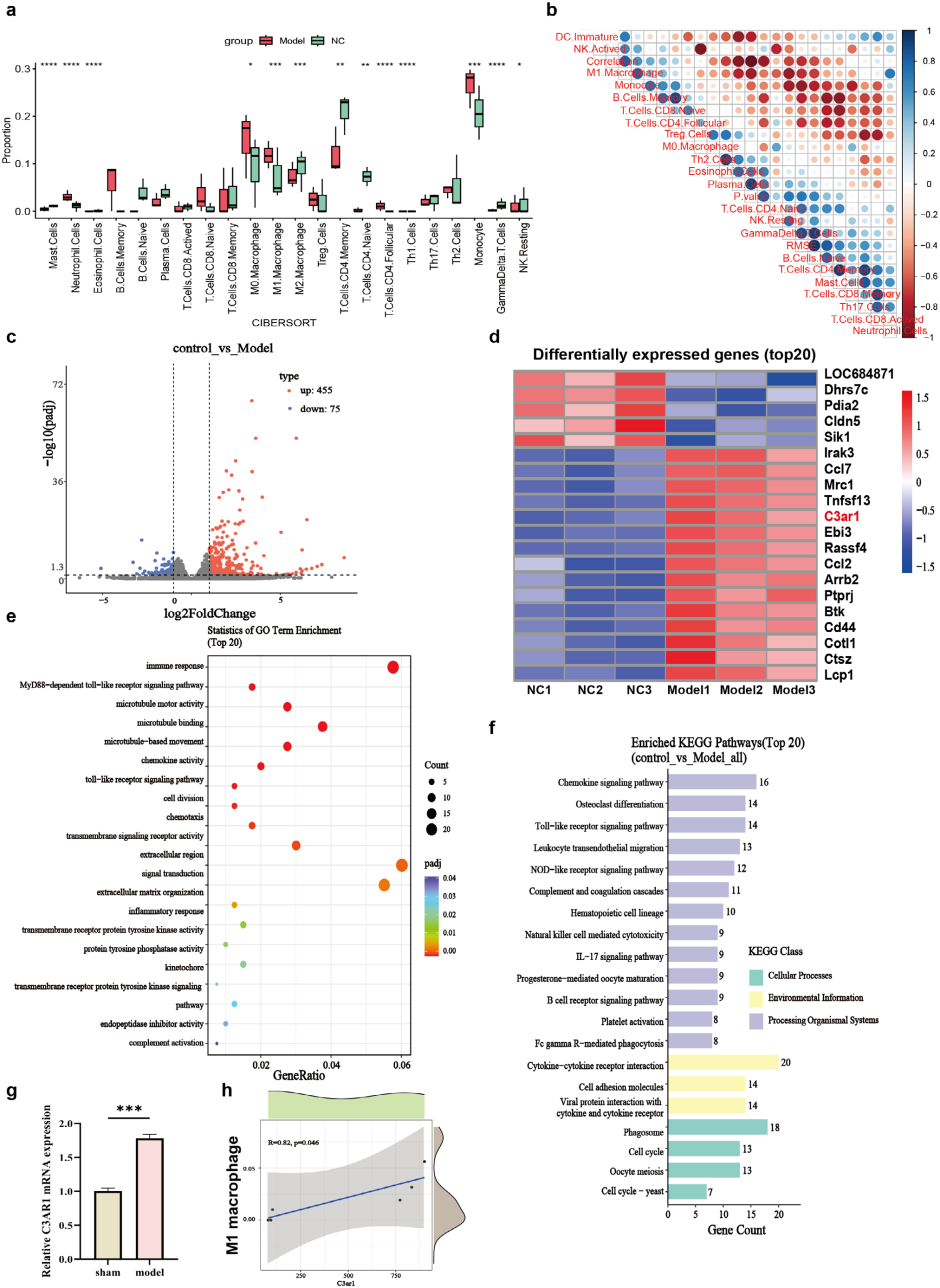
Myocardial immune microenvironment and c3ar1 expression in septic rats. (a) Relative proportions of 22 immune cell subtypes in myocardial tissues from sham‐operated (NC, green) and CLP‐induced septic (Model, red) rats, calculated using CIBERSORT algorithm. (b) Spearman correlation heatmap of immune cell populations. Blue indicates positive correlation, red indicates negative correlation. (c) Volcano plot of DEGs. Red dots: Upregulated genes; blue dots: Downregulated genes. (d) Heatmap showing expression patterns of top 30 DEGs. Red: Upregulated; blue: Downregulated. (e) enrichment analysis of DEGs (top 20 biological process terms). Dot size represents gene count; colour indicates adjusted *p*‐value. (f) KEGG pathway enrichment analysis of the DEGs (top 20 pathways). Bar length represents gene count; colour indicates pathway categories. (g) RT‐qPCR validation of C3aR1 mRNA expression in myocardial tissues (*n* = 5 per group). ***p < 0.001 vs. sham. (h) Spearman correlation analysis between C3aR1 expression and M1 macrophage. Each dot represents an individual sample.

To investigate immune changes in septic myocardium, we performed transcriptomic sequencing on myocardial tissues from CLP‐induced septic rats. Immune cell composition analysis using CIBERSORT revealed significant remodelling of the myocardial immune microenvironment in the sepsis group compared to controls (Figure 1a). Notably, the proportions of M0 and M1 macrophages, as well as neutrophils, were significantly increased, while anti‐inflammatory M2 macrophages were reduced. Correlation analysis also demonstrated a strong positive association between M1 macrophages and neutrophils (Figure 1b), suggesting coordinated pro‐inflammatory responses in septic myocardium.

Differential expression analysis identified 530 DEGs between groups (|log_2_FC| > 1, adjusted *p* < 0.05), with 455 upregulated and 75 downregulated genes (Figure 1c). Notably, the expression level of the complement C3 receptor gene C3ar1 was significantly increased (Figure 1d). GO enrichment analysis revealed that DEGs were primarily involved in inflammatory response, immune cell chemotaxis and necroptosis pathways (Figure 1e). KEGG pathway analysis further highlighted enrichment in NOD‐like receptor and Toll‐like receptor signalling pathways (Figure 1f), both critical for innate immune activation in sepsis.

RT‐qPCR validation confirmed a significant upregulation of C3ar1 mRNA expression in septic myocardial tissue (Figure 1g, *p* < 0.001). Importantly, correlation analysis based on transcriptomic data revealed a significant positive correlation between C3aR1 expression and M1 macrophage (Figure 1h, *R* = 0.82, *p* < 0.05). These findings identify C3aR1 as a potential key regulator of pro‐inflammatory immune responses in sepsis‐induced myocardial injury, prompting further functional investigation.

### 3.2 Cardiac‐Specific C3aR1 Knockdown Attenuates Sepsis‐Induced Myocardial Dysfunction

**FIGURE 2:**
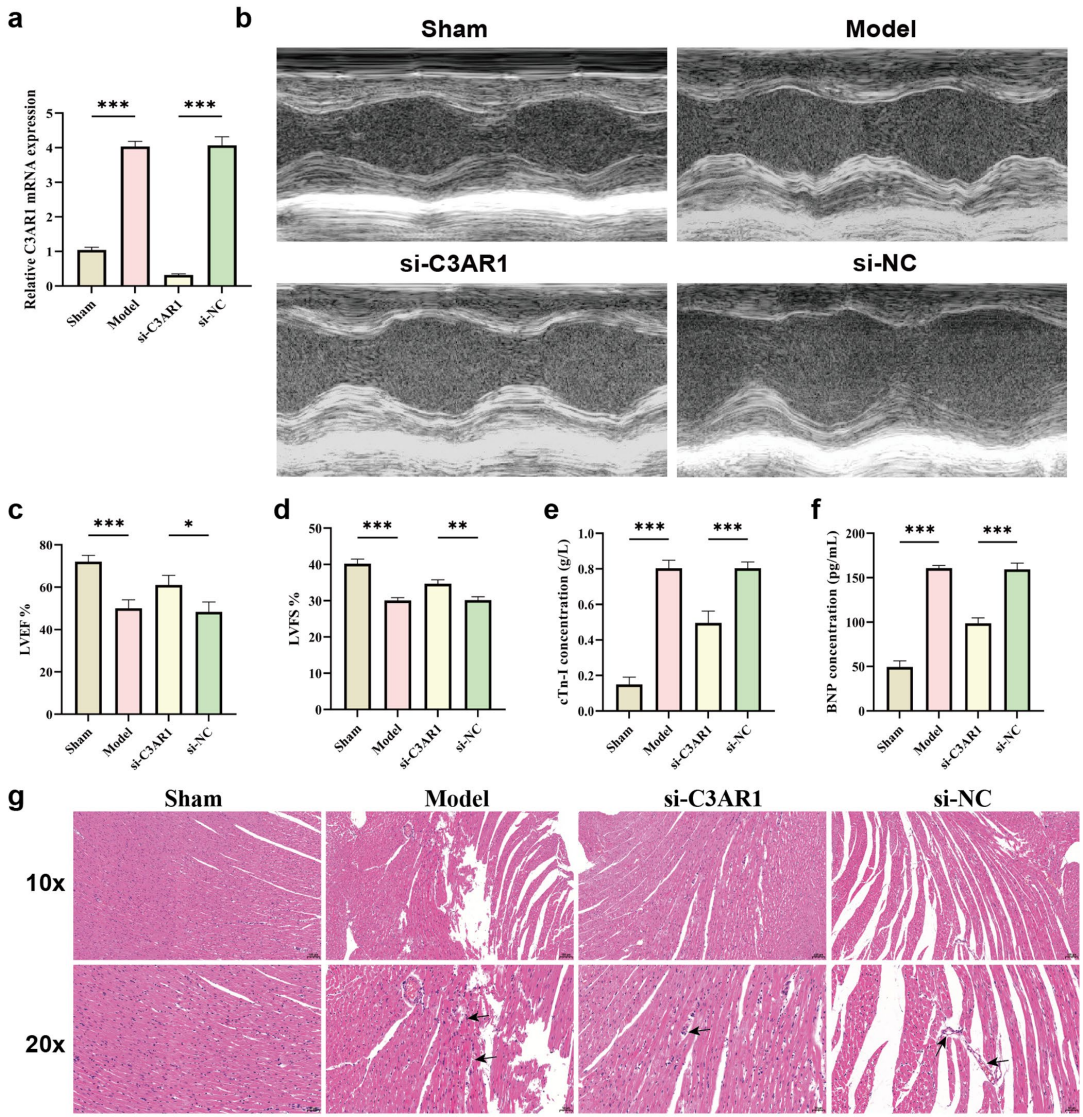
C3aR1 knockdown improves cardiac function and reduces myocardial injury in septic rats. (a) Relative mRNA expression of C3aR1 in rat myocardial tissue detected by RT‐qPCR. (b) Representative M‐mode echocardiographic images showing left ventricular wall motion. (c, d) Quantitative analysis of LVEF and LVFS. (e, f) Serum levels of cTnI and BNP measured by ELISA. (g) Representative H&E‐stained myocardial sections. Black arrows indicate inflammatory cell infiltration. Scale bar = 100 μm. *p < 0.05, **p < 0.01, ***p < 0.001. The same notation applies to the subsequent figures below.

To determine the functional role of C3aR1 in sepsis‐induced myocardial injury, we performed cardiac‐specific C3aR1 knockdown using rAAV9‐cTnT‐si‐C3aR1 via localised myocardial injection. RT‐qPCR confirmed effective knockdown, with C3aR1 mRNA levels significantly decreased in the si‐C3aR1 group compared to the Model group (Figure 2a, *p* < 0.001), while the si‐NC group showed no significant reduction.

Echocardiographic assessment at 24 h post‐CLP revealed severe cardiac dysfunction in septic rats (Figure 2b–d). Left ventricular ejection fraction (LVEF) and fractional shortening (LVFS) were markedly reduced in Model and si‐NC groups compared to Sham. In contrast, C3aR1 knockdown significantly improved cardiac function, with LVEF and LVFS increasing, indicating preservation of systolic function. Consistent with functional improvement, serum levels of myocardial injury markers, cardiac troponin I (cTnI) and brain natriuretic peptide (BNP), were substantially elevated in the Model and si‐NC groups, but significantly reduced in the si‐C3aR1 group (Figure 2e,f). Histological examination by H&E staining (Figure 2g) revealed extensive myocardial fibre disarray, necrosis and inflammatory cell infiltration in the Model and si‐NC groups. In contrast, the si‐C3aR1 group showed preserved myocardial architecture with markedly reduced necrotic areas and inflammatory infiltration. These results consistently demonstrate that C3aR1 plays a critical pathogenic role in sepsis‐induced myocardial injury and its inhibition confers significant cardioprotection.

### 3.3 C3aR1 Knockdown Suppresses Sepsis‐Induced Macrophage M1 Polarisation

**FIGURE 3:**
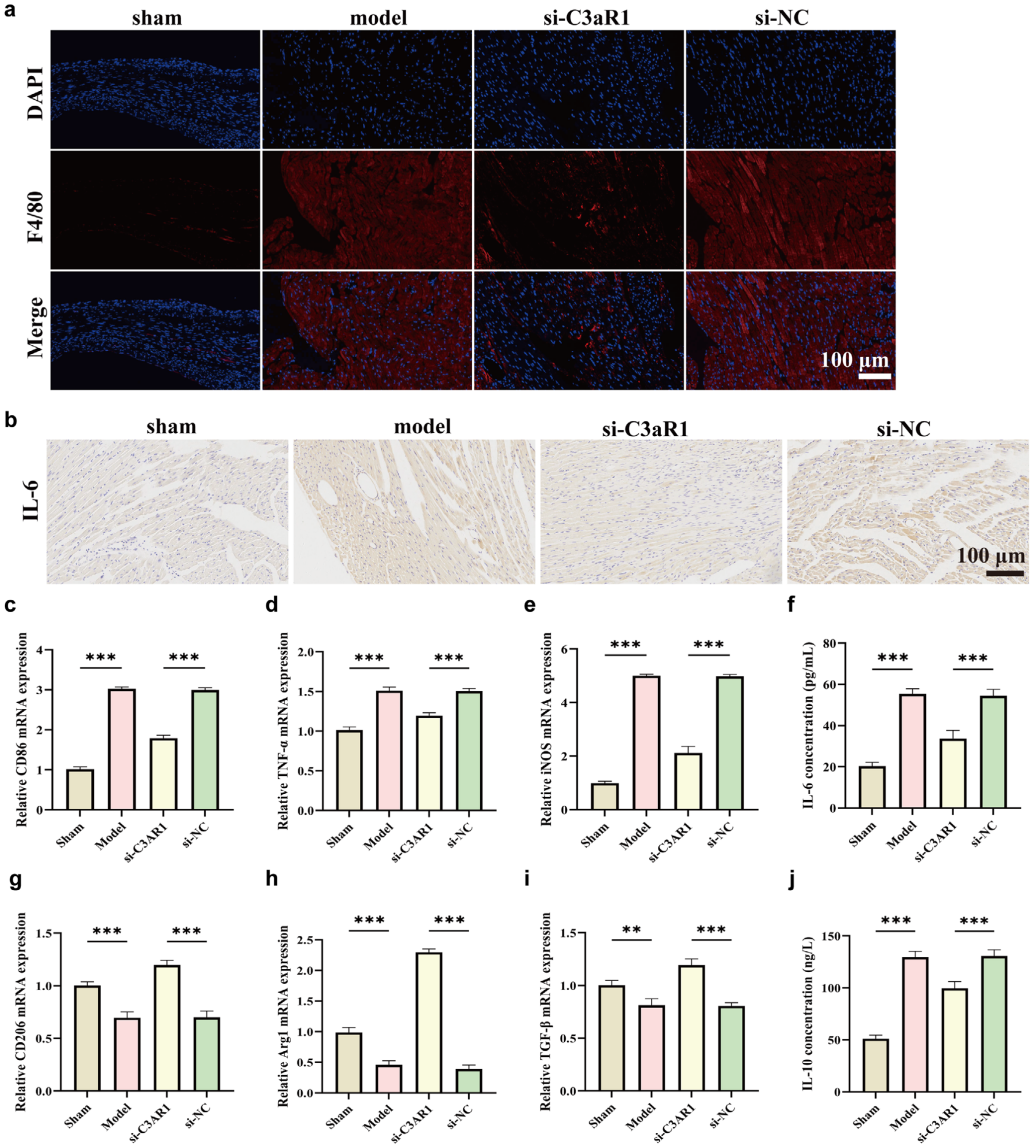
C3aR1 knockdown reverses sepsis‐induced macrophage M1 polarisation. (a) Immunofluorescence staining for F4/80 (red, macrophage marker) in myocardial tissues. Nuclei stained with DAPI (blue). Scale bar = 100 μm. (b) Immunohistochemical staining for IL‐6. (c–e) RT‐qPCR analysis of M1 macrophage markers: CD86 (c), TNF‐α (d) and iNOS (e). (f) ELISA measurement of IL‐6 levels in myocardial tissue homogenates. (g–i) RT‐qPCR analysis of M2 macrophage markers: CD206 (g), Arg1 (h) and TGF‐β (i). (j) ELISA measurement of IL‐10 levels in myocardial tissue homogenates.

Given the correlation between C3aR1 expression and M1 macrophage observed in transcriptomic data, we investigated whether C3aR1 regulates macrophage polarisation in vivo. Immunofluorescence staining for F4/80 (macrophage marker) revealed markedly increased macrophage infiltration in myocardial tissues of Model and si‐NC groups, which was significantly attenuated by C3aR1 knockdown (Figure 3a). Immunohistochemical analysis showed elevated IL‐6 expression in septic myocardium, consistent with a pro‐inflammatory milieu and this elevation was suppressed in the si‐C3aR1 group (Figure 3b).

To quantitatively assess macrophage polarisation, we measured mRNA expression of M1 and M2 markers by RT‐qPCR. In the Model and si‐NC groups, M1 markers (CD86, TNF‐α, iNOS) were significantly upregulated (Figure 3c–e), while the M2 markers (CD206, Arg1, TGF‐β) were downregulated (Figure 3g–i). In contrast, C3aR1 knockdown dramatically reversed this pattern, reducing M1 marker expression and restoring M2 marker expression to near‐Sham levels. Consistent with gene expression changes, ELISA measurements showed that pro‐inflammatory IL‐6 levels were elevated in the Model and si‐NC groups (Figure 3f), while anti‐inflammatory IL‐10 was reduced (Figure 3j). C3aR1 knockdown significantly reduced IL‐6 and increased IL‐10. These findings establish that C3aR1 critically regulates the balance between pro‐inflammatory M1 and anti‐inflammatory M2 macrophages in septic myocardium, with C3aR1 deficiency favouring an anti‐inflammatory phenotype.

### 3.4 C3aR1 Knockdown Attenuates Neutrophil Necroptosis and Chemokine Release

**FIGURE 4:**
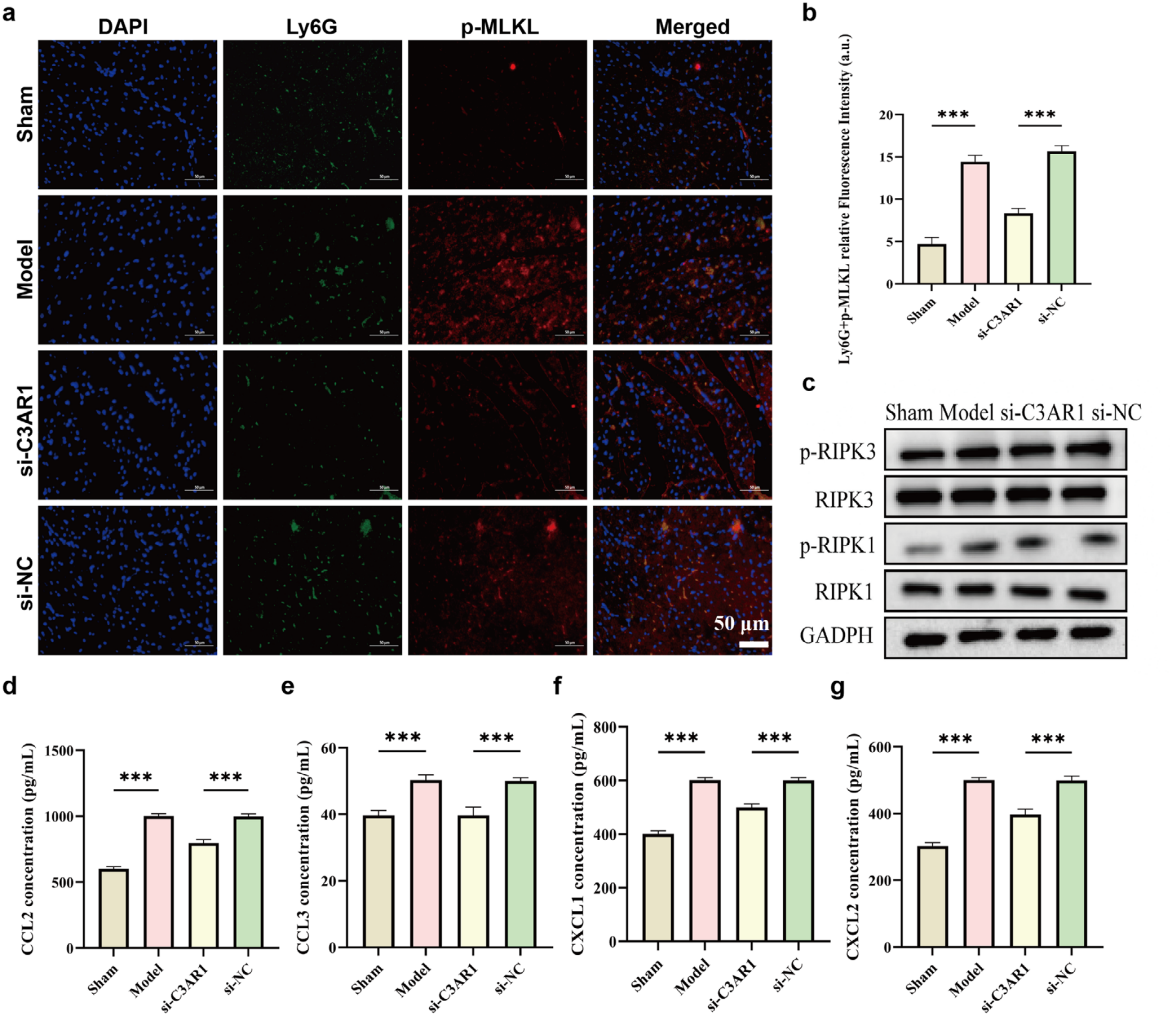
C3aR1 knockdown reduces neutrophil necroptosis and chemokine production in septic myocardium. (a) Representative immunofluorescence images showing co‐localisation of Ly6G (green, neutrophil marker) and p‐MLKL (red, necroptosis marker) in myocardial tissues. Nuclei stained with DAPI (blue). Scale bar = 50 μm. (b) Quantitative analysis of the fluorescence intensity of Ly6G^+^ *p*‐MLKL^+^ double‐positive cells. (c) Western blot analysis of necroptosis‐related proteins (p‐RIPK1, RIPK1, p‐RIPK3, RIPK3, p‐MLKL, MLKL) in myocardial tissues. (d–g) ELISA measurements of chemokines CCL2 (d), CCL3 (e), CXCL1 (f) and CXCL2 (g) in myocardial tissue homogenates.

Transcriptomic analysis revealed a strong positive correlation between M1 macrophage and neutrophil, suggesting potential crosstalk. To determine whether C3aR1‐mediated M1 polarisation influences neutrophil fate, we examined neutrophil necroptosis in myocardial tissues. Immunofluorescence double staining for Ly6G (neutrophil marker) and p‐MLKL (necroptosis marker) demonstrated robust co‐localisation in the Model and si‐NC groups (Figure 4a,b), indicating active neutrophil necroptosis. While this signal was markedly attenuated in the si‐C3aR1 group, suggesting inhibition of neutrophil necroptosis.

Western blot analysis of necroptosis pathway components confirmed these findings (Figure 4c). The expression of p‐RIPK1 and p‐RIPK3 (key events initiating necroptosis) was significantly increased in the Model and si‐NC groups. C3aR1 knockdown markedly reduced phosphorylation of RIPK1 and RIPK3. Given that necroptotic cells release damage‐associated molecular patterns and chemokines, we measured chemokine levels in myocardial tissue. ELISA revealed significantly elevated concentrations of CCL2, CCL3, CXCL1 and CXCL2 in the Model and si‐NC groups (Figure 4d–g). Whereas the levels of these chemokines were significantly decreased in the si‐C3aR1 group. These results demonstrate that C3aR1 promotes neutrophil necroptosis and subsequent chemokine release, likely through its effect on M1 macrophage polarisation.

### 3.5 C3aR1 Drives Macrophage M1 Polarisation via the TLR4/NF‐κB Pathway

**FIGURE 5:**
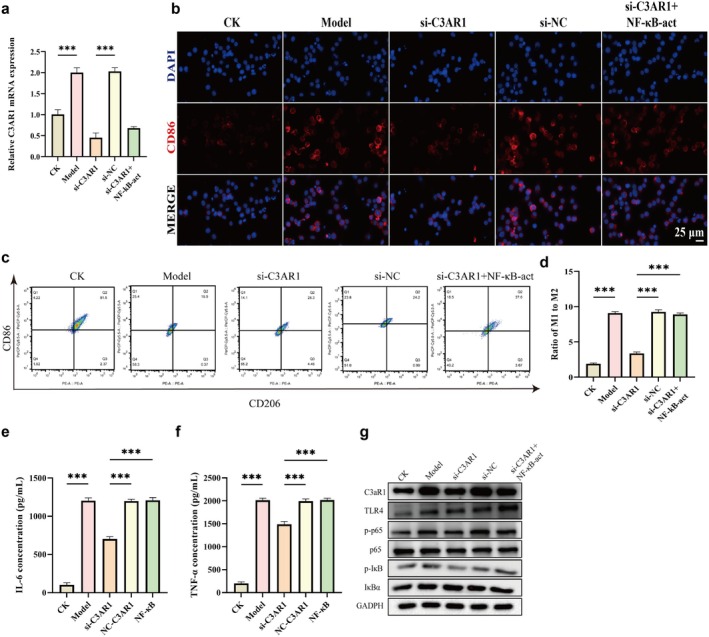
C3aR1 promotes M1 polarisation via TLR4/NF‐κB pathway. (a) RT‐qPCR analysis of C3aR1 mRNA expression in THP‐1 macrophages after indicated treatments. (b) Immunofluorescence staining for CD86 (green, M1 marker). Nuclei stained with DAPI (blue). Scale bar = 20 μm. (c) Representative flow cytometry plots showing M1 and M2 macrophage populations. (d) Quantitative analysis of M1/M2 ratio from flow cytometry. (e, f) ELISA measurements of IL‐6 (e) and TNF‐α (f) in culture supernatants. (g) Western blot analysis of C3aR1 and TLR4/NF‐κB pathway proteins (TLR4, p‐IκB, IκB, p‐p65 and p65).

To elucidate the molecular mechanism by which C3aR1 regulates M1 polarisation, we established an in vitro model using THP‐1‐derived macrophages. Given that KEGG enrichment analysis implicated Toll‐like receptor signalling, we focused on the TLR4/NF‐κB pathway. THP‐1 macrophages were transfected with si‐C3aR1 or si‐NC, then stimulated with LPS (100 ng/mL) and IFN‐γ (20 ng/mL) to induce M1 polarisation. RT‐qPCR results (Figure 5a) showed that LPS and IFN‐γ significantly upregulated C3aR1 expression (*p* < 0.001), which was effectively suppressed in the si‐C3aR1 group (*p* < 0.001). Immunofluorescence staining for CD86 (M1 marker) showed that LPS/IFN‐γ stimulation dramatically increased M1 polarisation, which was markedly suppressed by C3aR1 knockdown (Figure 5b). Flow cytometry revealed that the M1/M2 ratio was markedly elevated in the Model group compared to control (Figure 5c,d, *p* < 0.001). C3aR1 knockdown significantly reduced this ratio (*p* < 0.001 vs. Model), indicating reversal of M1 polarisation. Notably, treatment with NF‐κB activator 1 (NF‐κB‐act) partially reversed the effect of C3aR1 knockdown (*p* < 0.01 vs. si‐C3aR1), implicating NF‐κB in C3aR1‐mediated polarisation.

Consistent with these findings, ELISA showed that LPS/IFN‐γ stimulation increased IL‐6 and TNF‐α secretion (Figure 5e,f, *p* < 0.05). C3aR1 knockdown significantly reduced both cytokines, effects partially reversed by NF‐κB‐act. Western blot analysis of C3aR1 and TLR4/NF‐κB pathway components revealed that LPS/IFN‐γ stimulation upregulated C3aR1, TLR4, p‐IκB and p‐p65 (Figure 5g). Whereas C3aR1 knockdown significantly attenuated these changes. These results demonstrate that C3aR1 drives macrophage polarisation towards the M1 phenotype under septic conditions by activating the TLR4/NF‐κB pathway.

### 3.6 C3aR1–TLR4/NF‐κB Axis in Macrophages Triggers Neutrophil Necroptosis

**FIGURE 6:**
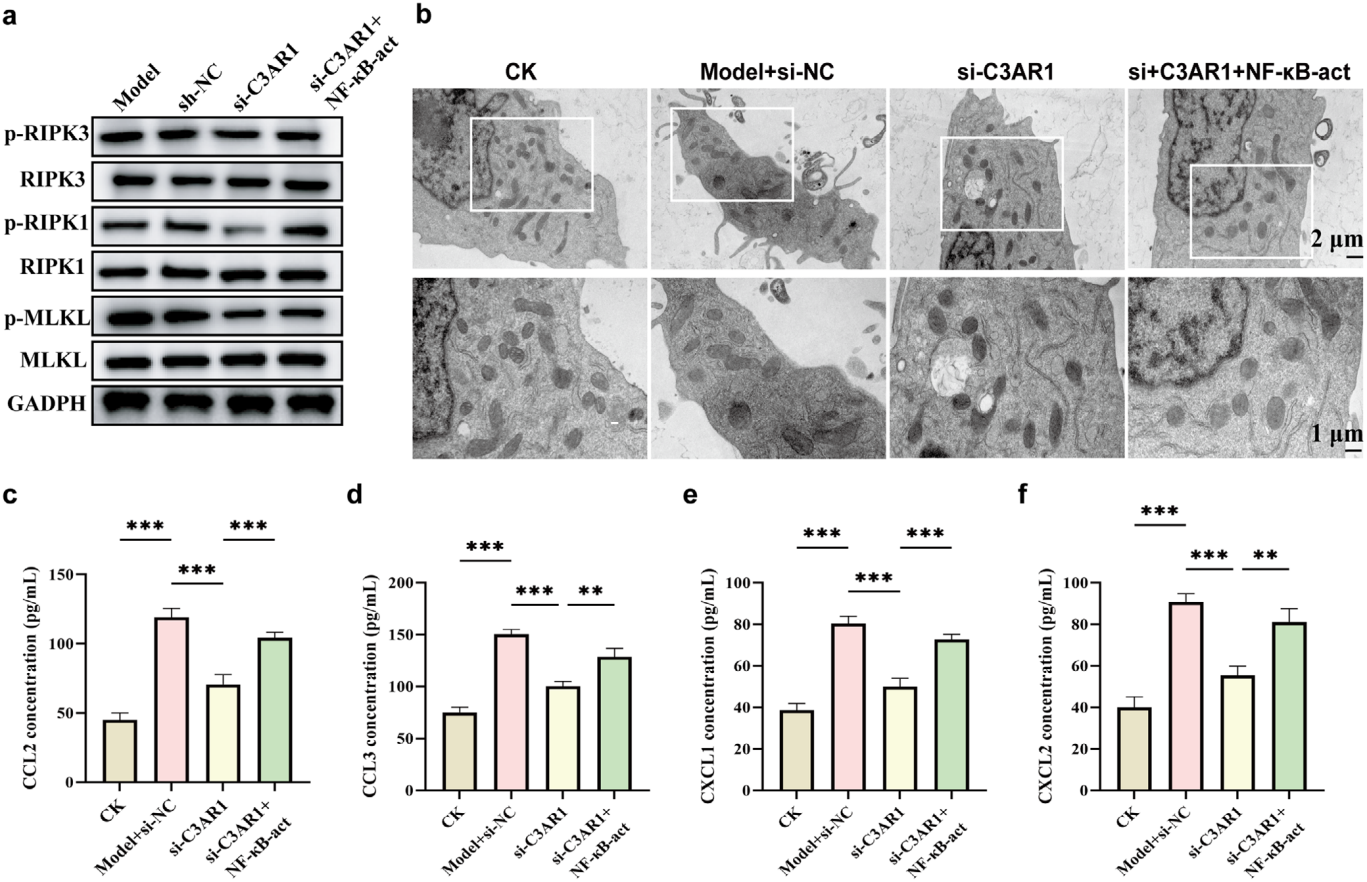
Macrophage C3aR1‐TLR4/NF‐κB axis induces neutrophil necroptosis. (a) Western blot analysis of necroptosis markers (p‐RIPK1, p‐RIPK3, p‐MLKL) in HL‐60 cells treated with conditioned media from indicated THP‐1 macrophage groups. (b) Representative TEM images illustrating the necroptotic morphology of HL‐60 cells. Lower panels show magnified views of the boxed regions, scale bar = 1 μm. (c–f) ELISA measurements of chemokines CCL2 (c), CCL3 (d), CXCL1 (e) and CXCL2 (f) in HL‐60 cell culture supernatants.

To determine whether C3aR1‐mediated M1 polarisation directly influences neutrophil necroptosis, we employed a conditioned medium approach. HL‐60 cells were cultured with conditioned media from macrophages subjected to different treatments. Western blot analysis revealed that conditioned medium from Model macrophages induced robust necroptosis in HL‐60 cells, evidenced by increased phosphorylation of RIPK1, RIPK3 and MLKL (Figure 6a). Notably, conditioned medium from si‐C3aR1 macrophages failed to induce necroptosis, with phosphorylation levels comparable to control. Importantly, NF‐κB activation restored the ability of si‐C3aR1 macrophages to induce neutrophil necroptosis, with phosphorylation levels approaching those observed in the Model group.

Transmission electron microscopy (TEM) confirmed these biochemical findings (Figure 6b). HL‐60 cells treated with Model macrophage supernatant exhibited characteristic necroptotic morphology: swollen mitochondria with disrupted cristae, plasma membrane rupture and cytoplasmic vacuolisation. In contrast, cells treated with si‐C3aR1 macrophage supernatant maintained intact cellular architecture with normal organelles. NF‐κB‐act treatment partially reversed this protection, restoring necroptotic features. ELISA measurement of chemokines in HL‐60 supernatants showed that Model macrophage supernatant induced significant release of CCL2, CCL3, CXCL1 and CXCL2 (Figure 6c–f). Supernatant from si‐C3aR1 macrophages failed to induce chemokine release, while NF‐κB‐act treatment partially restored this capacity. These results indicate that C3aR1 in macrophages, acting through TLR4/NF‐κB signalling, generates a pro‐necroptotic milieu that triggers neutrophil necroptosis and subsequent chemokine release.

### 3.7 Neutrophil Necroptosis Directly Injures Cardiomyocytes

**FIGURE 7:**
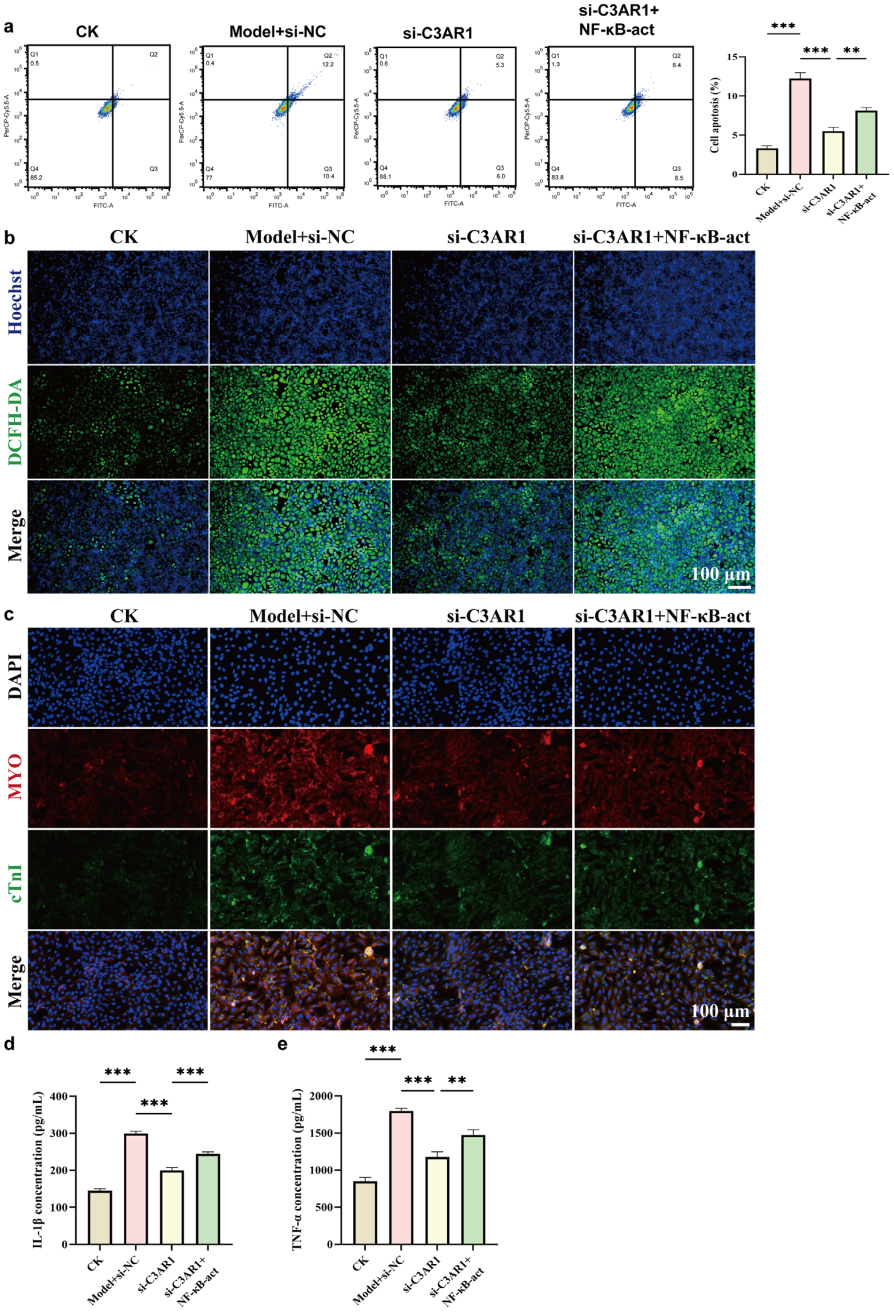
Neutrophil necroptosis directly injures AC16 cardiomyocytes. (a) Representative flow cytometry plots (left) and quantitative analysis (right) of AC16 cardiomyocyte apoptosis after co‐culture with HL‐60 neutrophils from indicated groups. (b) Representative fluorescence images showing ROS levels in AC16 cells, detected by fluorescent probe (blue: Hoechst‐stained nuclei; green: DCFH‐DA probe fluorescence). Scale bar = 100 μm. (c) Representative immunofluorescence images of myosin (MYO, red), cardiac troponin I (cTnI, green) and nuclei (DAPI, blue) in AC16 cells. Scale bar = 100 μm. (d, e) ELISA measurements of IL‐1β (d) and TNF‐α (e) in co‐culture supernatants.

To establish the final link in the pathogenic cascade, from macrophage C3aR1 activation to cardiomyocyte injury, we co‐cultured AC16 human cardiomyocytes with HL‐60 neutrophils (using Transwell inserts to prevent direct contact) under conditions where neutrophils were exposed to different macrophage supernatants as described above. Flow cytometry analysis of AC16 apoptosis revealed that co‐culture with neutrophils pre‐treated with Model macrophage supernatant significantly increased cardiomyocyte apoptosis (Figure 7a). Co‐culture with neutrophils from si‐C3aR1 macrophage supernatant dramatically reduced apoptosis, while NF‐κB‐act treatment partially reversed this protection.

Oxidative stress assessment using DCFH‐DA probe showed that co‐culture with Model‐treated neutrophils induced robust ROS production in AC16 cells (Figure 7b). ROS generation was markedly attenuated in the si‐C3aR1 group and partially restored by NF‐κB‐act. Immunofluorescence staining for cardiac‐specific markers revealed that co‐culture with Model‐treated neutrophils caused disrupted expression patterns of myoglobin (MYO) and cardiac troponin I (cTnI), with irregular distribution and reduced intensity in damaged cells (Figure 7c). These abnormalities were largely prevented in the si‐C3aR1 group but partially restored by NF‐κB‐act.

Finally, ELISA measurement of inflammatory cytokines in co‐culture supernatants showed that Model‐treated neutrophils induced significant release of TNF‐α and IL‐1β (Figure 7d,e). C3aR1 knockdown in the macrophage‐neutrophil cascade reduced these cytokines, effects partially reversed by NF‐κB‐act. Collectively, these results establish that C3aR1‐driven macrophage M1 polarisation triggers neutrophil necroptosis and these necroptotic neutrophils directly injure cardiomyocytes through multiple mechanisms, including apoptosis induction, oxidative stress and inflammatory cytokine release.

## 4 Discussion

Sepsis‐induced myocardial injury is an independent risk factor for mortality in septic patients [36]. Recent studies have revealed that disturbances in iron metabolism [37] and methylglyoxal‐induced capillary leakage [38] are also critical mechanisms in sepsis pathophysiology, highlighting the multifaceted nature of this condition. A hallmark of sepsis is the concurrent dysregulation of hyperinflammation and immunosuppression. Activated neutrophils and macrophages exacerbate organ damage by releasing inflammatory cytokines and forming neutrophil extracellular traps, while a persistent immune imbalance is linked to long‐term patient mortality [2, 39]. Excessive complement activation serves as a key initiator of the systemic inflammatory response. The complement receptor C3aR1, the primary effector molecule for C3a, is significantly upregulated in sepsis, suggesting its potential as a marker for disease severity. Although single‐cell RNA sequencing has identified a C3aR1+ macrophage subpopulation in cardiac tissues, its specific role and regulatory network in myocardial injury have remained unclear [31, 40].

This study demonstrates that C3aR1 deficiency protects against sepsis‐induced myocardial injury by modulating the immune microenvironment. Our transcriptomic analysis revealed a significant increase in M1 macrophages and neutrophils in the myocardium of septic rats, with a positive correlation between these cell types. C3aR1 expression was also markedly upregulated and associated with M1 macrophages. Functionally, interfering with C3aR1 in vivo reversed the CLP‐induced cardiac systolic dysfunction, pathological damage and elevated injury markers (cTn‐I, BNP). These findings align with the established role of the complement system in sepsis, where C3a binding to C3aR1 triggers downstream inflammatory signalling that exacerbates tissue damage [28].

During the early stage of sepsis, the ‘cytokine storm’ triggered by overactivated M1 macrophages is a significant contributor to tissue injury and the massive release of pro‐inflammatory cytokines is directly associated with pathological damage and mortality risk [41]. Our in vitro and in vivo results further elucidate the underlying mechanisms. C3aR1 deficiency bidirectionally regulates macrophage polarisation, suppressing M1 markers (CD86, iNOS, TNF‐α, IL‐6) and promoting M2 markers (CD206, Arg1, TGF‐β, IL‐10). This was accompanied by reduced neutrophil necroptosis, evidenced by lower expression of p‐RIPK1, p‐RIPK3 and p‐MLKL, fewer Ly6G/p‐MLKL double‐positive cells and diminished chemokine release. The phosphorylation of MLKL (p‐MLKL) is a crucial prerequisite for neutrophil necroptosis, leading to the release of damaging DAMPs like HMGB1, which amplifies the inflammatory response [42, 43, 44]. The pathological significance of this dual regulation is clear, as the M1/M2 macrophage imbalance is central to the dysregulated inflammation in sepsis [45]. The regulation of neutrophil cell death is therefore crucial for inflammation resolution [46]. Notably, immunofluorescence confirmed C3aR1 enrichment on F4/80‐positive macrophages in septic myocardial tissue, supporting the hypothesis that C3aR1 directly regulates macrophage function, consistent with findings in cardiac regeneration models [47].

Mechanistically, in THP‐1‐derived macrophages, C3aR1 deficiency significantly suppressed the TLR4/NF‐κB signalling axis, downregulating TLR4 and the downstream signalling molecules IκB, p‐IκB, p65 and p‐p65. This consequently attenuated M1 polarisation and pro‐inflammatory cytokine secretion, effects that were reversed by an exogenous NF‐κB activator. TLR4 is the primary receptor for LPS, initiating a MyD88‐dependent cascade that activates the IKK complex, leading to NF‐κB nuclear translocation and pro‐inflammatory gene transcription [48, 49]. This pathway is a key driver of multi‐organ injury in sepsis [50]. While previous research has established “crosstalk” between complement receptors and TLR pathways [51, 52], Our study specifically demonstrates that C3aR1 enhances the efficiency of TLR4 signalling, amplifying the NF‐κB‐mediated inflammatory response. This mechanism aligns well with the established paradigm of the TLR signalling pathway acting as a “core driver” regulating immune cell activation in the pathological state of sepsis [53], further corroborating the pivotal role of C3aR1 within the TLR4/NF‐κB‐mediated inflammatory regulatory network.

More importantly, this study identifies the C3aR1‐TLR4/NF‐κB axis as a key driver pathway in the ‘M1 macrophage polarisation—neutrophil necroptosis—cardiomyocyte injury’ vicious cycle. Pro‐inflammatory factors (IL‐6, TNF‐α) secreted by M1 macrophages induce necroptosis in HL‐60 cells, characterised by cell membrane rupture, cytoplasmic content release and upregulation of p‐RIPK1, p‐RIPK3, p‐MLKL and chemokines. This is consistent with the known mechanism where TNF‐α activates the RIPK1/RIPK3/MLKL pathway to trigger necroptosis [54, 55]. These necroptotic neutrophils then exacerbate AC16 cardiomyocyte injury through multiple pathways: releasing HMGB1 to activate caspase‐3 [56], secreting TNF‐α and IL‐1β to sustain NF‐κB activation [57] and generating ROS to damage mitochondria and contractile function [58]. Our co‐culture experiments validated these mechanisms, showing increased AC16 apoptosis and ROS, downregulated MYO and troponin and elevated TNF‐α and IL‐1β, consistent with Hu et al. [59]. This confirms that necroptotic neutrophils are key effector cells in sepsis‐induced myocardial injury. However, it is important to acknowledge that other forms of cell death, such as inflammasome‐mediated pyroptosis, may also contribute to sepsis‐induced organ dysfunction [60], suggesting a potential synergy among different cell death pathways in myocardial injury.

**FIGURE 8:**
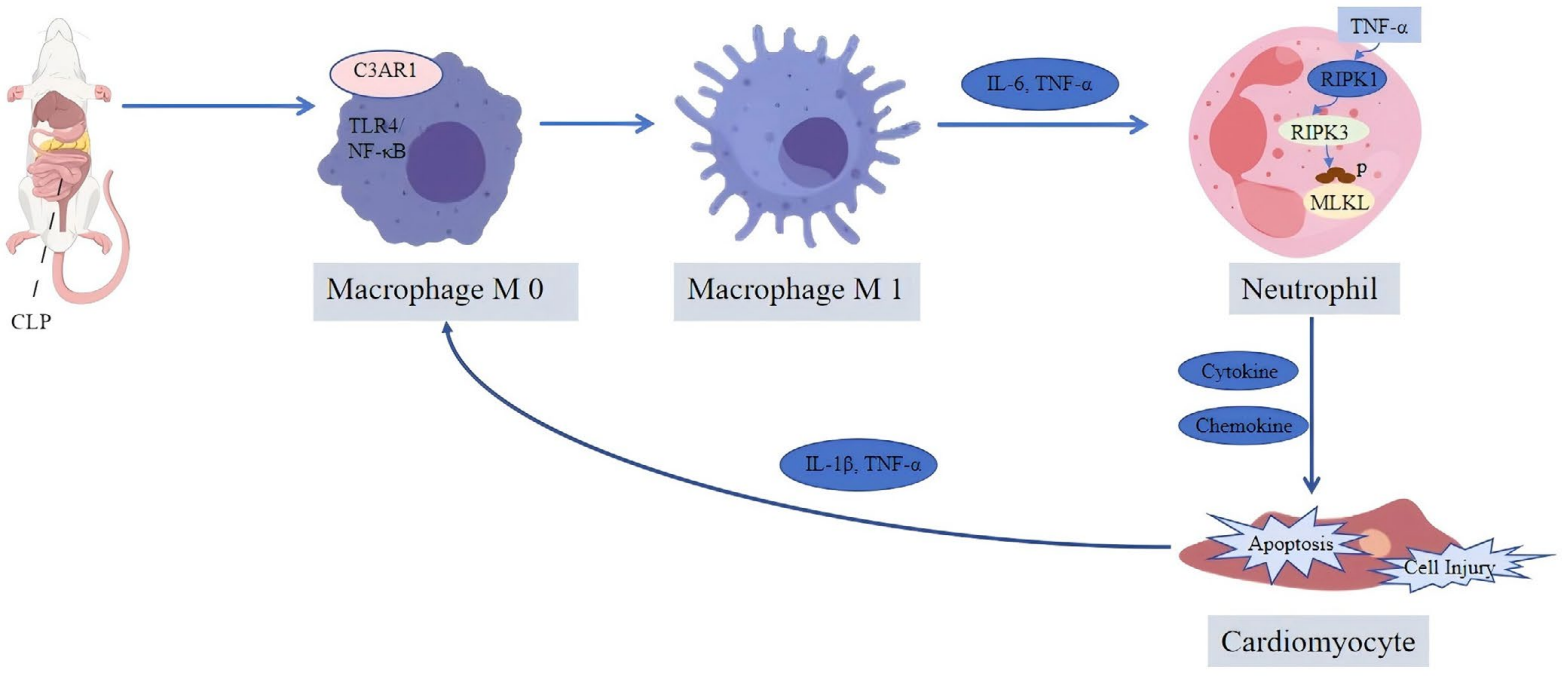
The C3aR1‐TLR4/NF‐κB signalling axis driving macrophage polarisation and neutrophil necroptosis in septic cardiomyopathy.

In summary, this study establishes macrophage C3aR1 as a key molecule driving sepsis‐induced myocardial injury by activating the TLR4/NF‐κB axis to promote M1 polarisation and subsequent neutrophil necroptosis. This inflammatory cascade provides a new theoretical basis for targeted immunomodulatory therapies (Figure 8). Nevertheless, this study has several limitations that warrant discussion. First, the reliance on the CLP bacterial peritonitis model, while robust, may not fully recapitulate the pathophysiology of other sepsis etiologies, such as viral sepsis (e.g., influenza), which can cause organ damage through different mechanisms like hematogenous dissemination to resident cells [61]. This limits the generalisability of our findings. Second, our research focused on the macrophage‐neutrophil axis, potentially overlooking the contributions of other immune cells within the complex septic microenvironment. Third, while effective, the lentiviral‐mediated C3aR1 knockdown used here carries inherent risks of off‐target effects and non‐specific immune responses. Future studies using macrophage‐specific conditional knockout mice would provide more precise genetic evidence. Finally, while our data strongly support the C3aR1‐TLR4/NF‐κB pathway as a central mechanism, alternative signalling cascades are likely involved. For instance, recent work has implicated EGFR/SRC/PI3K and NF‐κB/MAPK pathways in sepsis [62], suggesting C3aR1 may be one node in a complex regulatory network. Its relative contribution requires further elucidation through multi‐pathway comparative studies.

Future research should address these limitations by: (1) validating our findings in diverse preclinical models, including viral or endotoxemic models; (2) employing conditional knockout mice to definitively establish the cell‐specific role of C3aR1; (3) investigating the interplay between C3aR1 signalling and other inflammatory pathways (e.g., EGFR) to map the broader regulatory network; (4) and most importantly, translating these findings to the clinic by assessing the association between C3aR1 expression on circulating or cardiac macrophages and clinical outcomes in septic patients. The development of highly selective C3aR1 small‐molecule inhibitors or neutralising antibodies will be a crucial next step towards clinical translation.

## 5 Conclusion

This study demonstrates that macrophage C3aR1 plays a central role in sepsis‐induced myocardial injury by activating the TLR4/NF‐κB pathway, driving M1 polarisation and subsequent neutrophil necroptosis. These findings position C3aR1 as a promising therapeutic target. Future efforts should focus on developing selective C3aR1 small‐molecule inhibitors or neutralising antibodies to disrupt this pathogenic cascade. Additionally, evaluating C3aR1 expression levels on circulating or cardiac macrophages as a biomarker for early risk stratification in septic patients could inform clinical decision‐making. Validation in patient cohorts and macrophage‐specific conditional knockout models will be essential to facilitate clinical translation.

## Author Contributions

**Rubing Zhang:** data curation, formal analysis, visualization, project administration. **Jianbo Xu:** writing – review and editing, writing – original draft, data curation, validation. **Xinyue Zhao:** data curation, investigation, software. **Ziteng Cai:** methodology, project administration, visualization. **Chaonan Peng:** conceptualization, data curation, writing – review and editing. **Zhilei He:** data curation, formal analysis, validation. **Weiqun Wang:** supervision, conceptualization, data curation.

## Funding

This work was supported by the Weiqun Wang. Youth Program of Open Research Project in State Key Laboratory of Neurology and Oncology Drug Development, China (SKLSIM #x2010; F #x2010; 2024120), the Weiqun Wang. Provincial Natural Science Foundation Joint Cultivation Project of Heilongjiang xxxFF0C; China (PL2024H012), the Xinyue Zhao. Basic Scientific Research Expenses Scientific Research Projects of Universities in Heilongjiang province, China (2023 #x2010; KYYWF #x2010; 0595), the East Pole team project of Jiamusi University, Heilongjiang, China (DJXSTD202405) and the 2025 Annual Xiaogan City Natural Science Plan Project, China (XGKJ2025010061).

## Ethics Statement

Animal experiments were approved and performed in accordance with the guidelines of the Animal Experimentation Ethics Committee of Hanchuan People's Hospital (20250415HCRY‐003).

## Conflicts of Interest

The authors declare no conflicts of interest.

## Data Availability

The data that support the findings of this study are available on request from the corresponding author. The data are not publicly available due to privacy or ethical restrictions.
